# Enhanced T Cell Responses Induced by a Necrotic Dendritic Cell Vaccine, Expressing HCV NS3

**DOI:** 10.3389/fmicb.2020.559105

**Published:** 2020-11-24

**Authors:** Zelalem A. Mekonnen, Makutiro G. Masavuli, Wenbo Yu, Jason Gummow, Dawn M. Whelan, Zahraa Al-Delfi, Joseph Torresi, Eric J. Gowans, Branka Grubor-Bauk

**Affiliations:** ^1^Viral Immunology Group, Discipline of Surgery, Basil Hetzel Institute for Translational Medicine, University of Adelaide, Adelaide, SA, Australia; ^2^Centre for Cancer Biology, University of South Australia, Adelaide, SA, Australia; ^3^Gene Silencing and Expression Laboratory, Robinson Research Institute, The University of Adelaide, Adelaide, SA, Australia; ^4^Department of Microbiology and Immunology, The Peter Doherty Institute for Infection and Immunity, University of Melbourne, Melbourne, VIC, Australia

**Keywords:** necrosis, HCV, cell mediated immunity, cross presentation, vaccine, hepatitis (C) virus

## Abstract

A vaccine that induces potent, broad and sustained cell-mediated immunity, resulting in effective memory has the potential to restrict hepatitis C (HCV) virus infection. Early, multi-functional CD4^+^ and CD8^+^ T cell responses against non-structural protein 3 (NS3) have been associated with HCV clearance. Necrotic cells generate strong immune responses and represent a major antigenic source used by dendritic cells (DC) for processing and presentation, but there is conflicting evidence as to their immunogenicity in vaccination. Immunization with DC loaded with viral antigens has been done in the past, but to date the immunogenicity of live vs. necrotic DC vaccines has not been investigated. We developed a DC2.4 cell line stably expressing HCV NS3, and compared the NS3-specific responses of live vs. necrotic NS3 DC. Vaccination of mice with necrotic NS3 DC increased the breadth of T-cell responses and enhanced the production of IL-2, TNF-α, and IFN-γ by effector memory CD4^+^ and CD8^+^T cells, compared to mice vaccinated with live NS3 DC. A single dose of necrotic NS3 DC vaccine induced a greater influx and activation of cross-presenting CD11c^+^ CD8α^+^ DC and necrosis-sensing Clec9A^+^ DC in the draining lymph nodes. Furthermore, using a hydrodynamic challenge model necrotic NS3 DC vaccination resulted in enhanced clearance of NS3-positive hepatocytes from the livers of vaccinated mice. Taken together, the data demonstrate that necrotic DC represent a novel and exciting vaccination strategy capable of inducing broad and multifunctional T cell memory.

## Introduction

In the majority (70–80%) of infected individuals hepatitis C (HCV) infection leads to chronic persistence of the virus, resulting in progressive, and often fatal liver disease and cancer. The WHO recently reported that the rate of new HCV infections which is 1.75 million annually, outpaces treatment and cure, setting back the goal to eliminate HCV by 2030 (Hill et al., 2017; WHO, 2017). HCV therapeutic cure, directly acting antivirals (DAAs), does not prevent reinfection or eliminate residual cancer risk. Therefore, the burden of chronic disease remains high. An estimated 71 million people world-wide are chronically infected with HCV, with as many as 50% unaware of their infection status. Overall, it is estimated that only 20% of infected individuals get diagnosed and only 2.9% are treated with DAAs (Sandmann et al., 2019; WHO, 2019). Furthermore, successful treatment with DAAs does not prevent re-infection and increasing evidence suggests that DAA failure can lead to the evolution of drug-resistant virus (Zeuzem et al., 2017; Smith et al., 2019), suggesting that even the best treatment options are unable to eradicate HCV. For this reason, developing a HCV vaccine to complement therapy is a public health priority in order to reduce the number of new HCV infections.

Developing vaccines for persistent infections remains a global challenge. A rational path for HCV vaccine design is to achieve immune responses that mimic those elicited during the resolution of acute HCV infection. Elimination of acute HCV infection requires strong T cell-mediated immunity (CMI) and broadly neutralising antibodies (NAb; Lechner et al., 2000; Thimme et al., 2001, 2002; Bowen and Walker, 2005). Development of vaccines that induce NAb against HCV has proved elusive for several reasons, mainly because current strategies fail to induce the extensive somatic hypermutations required to generate broad NAb (Edwards VC et al., 2012).

Human and chimpanzee studies have demonstrated that broad and sustained HCV-specific CD8^+^ and CD4^+^ T cell responses against highly conserved non-structural (NS) proteins, including NS3 result in clearance of acute HCV (Liang, 2013). Antibody-mediated depletion of CD8^+^ and CD4^+^ T cells in chimpanzees lead to virus persistence (Grakoui et al., 2003; Shoukry et al., 2003). Furthermore, T cell responses were responsible for the persistent immunity after clearance of primary infection, resulting in the reduction in the level and duration of viremia after reinfection. Viral clearance was observed in 83% of reinfected patients, compared to 25% in primary infection and was associated with a rapid T cell memory recall (Osburn et al., 2010).

Therefore, it is becoming apparent that an effective HCV vaccine strategy should induce strong CMI, targeting conserved viral proteins, such as the NS proteins (Post et al., 2004; Grubor-Bauk et al., 2015; Gummow et al., 2015; Wijesundara et al., 2018; Mekonnen et al., 2019). Among the NS proteins, NS3 is an appropriate vaccine candidate as it is one of the most genetically conserved HCV antigens across all genotypes, and studies have shown a direct correlation between NS3-specific immune responses and resolution of acute infection (Diepolder et al., 1995, 1997; Schulze zur Wiesch et al., 2005).

Professional antigen presenting cells (APC), such as dendritic cells (DC), process and present antigens to prime naïve T lymphocytes, thus initiating adaptive immune responses (Steinman, 2012). DC which are not directly infected with a pathogen are capable of capturing, processing, and presenting exogenous antigens (Ag) onto MHC class I molecules and presenting these in a context of co-stimulatory molecules, a process known as cross-presentation [reviewed in Joffre et al. (2012)]. Cross presentation is essential for the initiation of Ag-specific cytotoxic CD8^+^ T cell responses and can be a used as a valuable tool to enhance vaccine responses. Although direct presentation of epitopes results from endogenous expression of the Ag in DC, as DC represent a rare population of cells, it is likely that cross-presentation of exogenous Ag is a more common pathway to induce immunity to vaccines which do not directly target DC [reviewed in Heath et al. (2004)]. Thus, successful vaccine strategies must target DC directly and/or otherwise facilitate cross-presenting DC to endocytose the immunogen.

*In vivo*, necrotic cells represent major sources of exogenous Ag used by DCs for cross-presentation, as they release products that influence Ag processing and presentation, such as damage associated molecular patterns (DAMPs; Matzinger, 1994; Shi et al., 2003; Gamrekelashvili et al., 2015). These include heat shock proteins (HSP), uric acid, high mobility group box 1 protein (HMGB1), genomic DNA and F-actin, which act as natural adjuvants (Sauter et al., 2000; Shi et al., 2003; Rock et al., 2011; Ahrens et al., 2012). DAMPs bind to pathogen recognition receptors (PRR) expressed in APC including DCs, resulting in maturation and up-regulation of T cell costimulatory molecules. These mature DC then migrate to draining lymph nodes (LN) where they prime naïve T cells and initiate adaptive T cell immunity.

In the past, studies have shown that freeze-thawed necrotic tumor cells failed to induce Ag-specific CD8^+^ T cell responses, despite the release of DAMPs (Scheffer et al., 2003; Casares et al., 2005). However, more recently two cellular peptidases released by necrotic cells, dipeptyl peptidase 3 (DPP-3), and thimet oligopeptidase (TOP-1), have been shown to control CD8^+^ T cell priming by proteasomal degradation and thus block Ag cross-presentation (Gamrekelashvili et al., 2013). Provided these peptidases were inactivated by heat, trypsin treatment or gene knockdown they were no longer able to degrade oligopeptides, resulting in T cell activation.

The inference from these observations is that heat-induced necrosis of antigen-positive cells will represent an important component of an effective immunization strategy, and we explored this possibility by immunization of mice with heat-induced necrotic DCs expressing NS3.

We developed a mouse DC (DC2.4) cell line, stably expressing HCV NS3 (genotype 1b). Vaccination with heat-induced necrotic NS3 DC resulted in a greater influx of necrosis-sensing, cross-presenting CD11c^+^ CD8α^+^ Clec9A^+^ DC in the LN draining the site of injection at early time points post-vaccination compared to mice vaccinated with live cells. This in turn, resulted in broader and enhanced effector memory NS3-specific CD8^+^ and CD4^+^ T cell responses and accelerated NS3 antigen clearance from the livers of vaccinated mice.

Collectively, our data suggest that necrotic cell vaccines encoding HCV NS3 merit further study in the context of future prophylactic HCV CMI-based vaccines.

## Materials and Methods

### Lentiviral Vector Construction and Packaging

The codon optimized HCV genotype 1b (g1b) NS3 gene was synthesized (Gene Art, Germany) and a third-generation lentiviral vector encoding this protein was generated with Gateway technology. In this system, expression of NS3 was under the control of the EF-1α promoter and the marker gene (GFP) was expressed from the encephalomyocarditis virus (EMCV) internal ribosome entry site (IRES) [as described in Brown et al. (2010)]. To generate a NS3 g1b lentiviral stock, HEK293T cells were co-transfected with 12.5 μg of the NS3 transfer vector (plvEIG), 7.5 μg of Gag/Pol (Δ8.2), 6.25 μg of Rev (pRSV-Rev), and 3.75 μg of Env (pCMV-VSV-G), using Lipofectamine LTX reagent (Life Technologies, Australia) and Opti-MEM (Life Technologies) reduced serum medium, in accordance with the manufacturer’s protocol. The next day, the medium was replaced with fresh RPMI and 48 h later the supernatant was collected, filtered (0.45 μm), and used immediately or stored at −70°C.

### Lentiviral Transduction and Establishment of NS3 DC2.4 Stable Cell Line

Wild type DC2.4 and the NS3 DC2.4 expressing stable line were cultured in RPMI 1640 medium supplemented with 100 U/ml penicillin, 100 μg/ml streptomycin, 2 mM L-glutamine, 10% FBS, 50 μM β-mercaptoethanol, 1 mM sodium pyruvate, and 10 mM HEPES buffer. For lentiviral transduction of the wild type DC2.4 cell line, the medium from cells in a 6-well plate was replaced with 3 ml of viral supernatant containing 8 μg/ml Polybrene. NS3-GFP-positive DC2.4 cells were sorted by flow cytometry (FACSAria; BD Biosciences, San Jose, CA, United States) until purity reached 93.6%.

### NS3 Immunofluorescence

To detect the expression NS3, wild type DC2.4 and NS3 DC2.4 cells were fixed with 4% formalin (Sigma Aldrich) for 20 min, permeabilized with methanol at -20°C then blocked in 2.5% BSA (Sigma Aldrich) in PBS. The primary antibody, mouse anti-HCV NS3 gt1b (Virostat) diluted in 1% BSA, 0.3% Triton X-100 (Sigma Aldrich) in PBS, was added and incubated overnight at 4°C. After washing, the cells were then incubated for 1 h with fluorophore-conjugated secondary antibody (1:300) anti-mouse – Cy5 (Life Technologies). Cells were visualized by fluorescent microscopy (Zeiss LSM-700).

### Induction of Necrosis

Live NS3 DC2.4 cells were resuspended in sterile PBS at a concentration of 1 × 10^7^/ml. The cells were heated to 63°C for 30 min to induce necrosis. Necrosis was confirmed by trypan blue staining.

### Animals and Immunization

Female C57BL6 mice were obtained from the University of Adelaide, Animal Laboratory Services or from the Animal Resource Centre. Mice were housed in the Women’s and Children’s Hospital (Adelaide, Australia) animal house and all experimental protocols were approved by the Women’s and Children’s Health Network and the University of Adelaide Animal Ethics Committee. Animal experiments were carried out according to the Australian Code of Practice for the care and use of animals for scientific purposes. Female C57BL/6 (6–8 weeks old) were immunized subcutaneously with 10^6^ live or necrotic wild type DC2.4 or NS3 DC2.4 cells in 100 μl of sterile PBS. In the standard prime-boost regimen, mice were vaccinated with the same dose at one-week intervals. 2 weeks after the final vaccination, unless otherwise indicated, mice were euthanized, and spleens were collected for analysis.

### IFN-γ ELISpot Assay

NS3-specific cellular immune responses were assessed by IFN-γ ELISpot assay as we described (Grubor-Bauk et al., 2015, 2019; Masavuli et al., 2019). Briefly, multiscreen-IP HTS plates (Millipore) were coated with anti-mouse IFNγ (clone AN18, MabTech) and secreted IFNγ was detected with anti-mouse IFNγ -biotin (clone R4-6A2, MabTech), streptavidin-AP (Sigma Aldrich) and SigmaFast BCIP/NBT (Sigma Aldrich). The 98-peptide array spanning the entire NS3 protein (strain J4L6S, genotype 1b) was obtained from the National Institutes for Health Bio Defense and Emerging Infectious Research Resources Repository, NIAID, National Institutes of Health United States.

Individual peptides overlap by 11–12 amino acids and detailed information on their length and sequence is provided by BEI Resources^1^. The NS3 peptides were divided into three pools, each containing 29–31 individual peptides. Two C57BL/6 (H-2b) epitopes, were described previously (Mikkelsen et al., 2011) and were used as the immunodominant pool to stimulate splenocytes in ELISpot and ICS. All peptides were used at a final concentration of 4 μg/ml and splenocytes were stimulated for 18–20 h at 37°C. Splenocytes stimulated with PHA (5 μg/ml) were used as a positive control and un-stimulated splenocytes as a negative (background) control. The spots were counted automatically using an ELISPOT reader (AID GmbH, Germany).

### Flow Cytometry

Multi-color intracellular cytokine staining (ICS) was performed on splenocytes re-stimulated with immunodominant NS3 peptides for 12 h in the presence of protein transport inhibitor (BD GolgiStop^TM^). Staining was performed with BD FACS Cytofix/Cytoperm and BD anti-mouse antibodies (CD3-PercP-Cy5.5, CD8-APC-Cy7, CD44-APC, IL-2-FITC, IFNγ –PeCy7, TNFα-PE, and BD Biosciences). The cells were analyzed on a BD FACS Canto II flow cytometer using the gating strategy we described previously (Gargett et al., 2014; Gummow et al., 2015). Briefly, splenocytes were gated on the lymphocyte population, followed by doublet discrimination and then gated on CD3^+^, CD44^*high*^ cells and finally CD4^+^ or CD8^+^ cells to assess the frequency effector memory CD4^+^ or CD8^+^ T cells. Within the CD44^high^ CD4^+^ or CD44^high^ CD8^+^ T cell populations single (IFNγ, TNFα, or IL-2), double (IFNγ and TNFα), and triple cytokine positive cells were identified (IFNγ, TNFα, and IL-2).

Dendritic cells staining was performed on cell suspensions obtained from the axillary draining LN using the following antibodies CD3-PerCP-Cy5, CD8a-APC-Cy7 (BD Bioscience), CD11c-PeCy7, CD86-PE, MHCII-FITC (EBioscience), and Clec9A-PE antibody from Miltenyi Biotec and analyzed as we described previously (Gargett et al., 2014; Garrod et al., 2014). Briefly, purified lymphocytes cells were gated on CD3^–^ cells (non T cells), and then on CD11c^*high*^ MHCII^+^ cells (DC), CD11c^*high*^ MHCII^+^ CD8a^+^ cells (cross-presenting CD8a^+^ DC) and CD11c^*high*^ MHCII^+^ CD8a^+^ Clec9A^+^ (necrosis-sensing Clec9a^+^ DC) or CD11c^*high*^ MHCII^+^ CD8a^+^ CD86^+^ (activated cross presenting CD8a DC). The results were analyzed with FlowJo X.0.7 software (Ashland, OR, United States).

### T Cell Proliferation Studies

Proliferation of CD8^+^ and CD4^+^ T cells was assessed by flow cytometry after CFSE staining. Splenocytes from vaccinated mice were labeled with 10 μM CFSE (CellTrace CFSE Cell Proliferation Kit protocol; Life Technologies) as per the manufacturer’s instructions and stimulated *in vitro* with 4 μg/mL immunodominant NS3 peptides or left unstimulated. After 5 days, CFSE-labeled splenocytes were surface stained with CD3-PerCP-Cy5.5, CD4-eFluor450, and CD8-APC-Cy7 (all from eBioscience), followed immediately by Hoechst staining prior to analysis by flow cytometry. Proliferative responses were measured by CFSE dilution assay of cell proliferation as we described previously (Gargett et al., 2014; Garrod et al., 2014; Gummow et al., 2015). The results were analyzed with FlowJo X.0.7 software (Ashland, OR, United States).

### Mouse Hydrodynamic Challenge

Female C57BL/6 mice were vaccinated with 10^6^ live or necrotic NS3 DC2.4 s.c. (standard prime-protocol) and challenged 2 weeks later by hydrodynamic injection of 20 μg of pNFS plasmid in TransIT-QR HD delivery solution (Mirus) in a volume of 1/10 body weight. Unvaccinated mice were challenged as a control. Hydrodynamic injection was performed as we described (Yu et al., 2014). In the pNFS plasmid used to challenge mice, NS3/4A protein expression is controlled by the mouse albumin promoter/α-fetoprotein enhancer ensuring hepatocyte-specific expression of HCV NS3/4A. Introns 1 and 2 were included to optimize expression. Secreted alkaline phosphatase (SEAP) is encoded as a fusion protein, preceded by the FMDV2A protease which is designed to self-cleave and release SEAP co-translationally. Any mouse which failed to receive the full challenge volume was excluded from further analysis. Serum samples were collected from the challenged mice at different time-points and levels of SEAP were measured using the Phospha-light kit (Applied Biosystems) in 96 well flat bottom white microplates (GreinerBioOne; Yu et al., 2014).

### Statistical Analysis

Data are presented as means ± the standard errors of the mean (SEM). Statistical methods including unpaired Mann–Whitney test, Kruskal–Wallis *H* test and log-rank (Mantel-Cox) test were used as necessary, with *P* ≤ 0.05 (^∗^), *P* ≤ 0.01 (^∗∗^), and *P* ≤ 0.001 (^∗∗∗^) considered significant. Analysis was performed using GraphPad Prism version 6.00 for Windows (GraphPad Software, La Jolla, CA, United States) with assistance from Dr. Stuart Howell from the Data Analysis and Management Centre, University of Adelaide.

## Results

### Necrotic NS3 DC2.4 Are More Immunogenic Than Live NS3 DC2.4 and Increase the Breadth of NS3-Specific T Cell Responses in Vaccinated Mice

Due to their central role in T cell activation, vaccination strategies involving DCs have been used in the past in patients suffering from cancer or from viral infections such as HCV and human immunodeficiency virus (Lu et al., 2004; Gowans et al., 2010; Palucka and Banchereau, 2012). In the case of HCV, overexpression of structural gene products such as core and envelope has been reported to interfere with the immunostimulatory functions of DC, but overexpression of NS3 had no effect on DC maturation (Sarobe et al., 2003). We transduced murine DC2.4 cells with a lentivirus encoding codon optimized NS3 (genotype 1b: gtb1b), under the control of the EF-1α promoter, and GFP controlled by the IRES from EMCV (Supplementary Figure 1A). Constitutive and stable expression of NS3 was shown by immunofluorescence, using untransduced, wild type, DC2.4 cells as negative controls (Supplementary Figure 1B). Using fluorescence-activated cell sorting, NS3 positive DC2.4 cells were purified and sorted on the basis of their GFP expression. The final purity of NS3 DC2.4 cells was 93.6%, as confirmed by flow cytometry (Supplementary Figure 1C). Similar to the overexpression of NS3 in DC derived from BALB/c and C57BL/6 mice (Zabaleta et al., 2008), overexpression of NS3 in DC2.4 had no significant effect on the expression of CD80, CD86, and CD11c, when compared to wild type DC2.4 cells (Supplementary Figure 1D). To induce heat-shock necrosis NS3 DC2.4 cells were heated to 63°C for 30 min and necrosis was confirmed by trypan blue staining.

**FIGURE 1:**
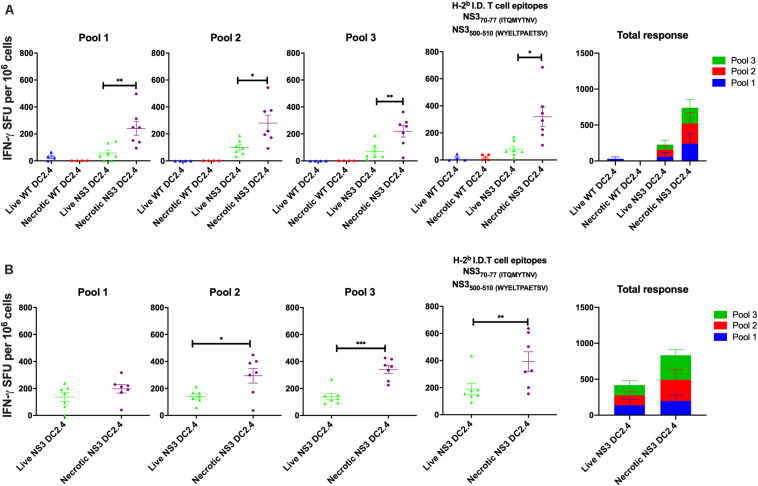
Necrotic NS3 DC2.4 cells are more immunogenic than live NS3 DC2.4 cells in C57BL/6 mice. Mice were vaccinated twice, at 2 week intervals with 10^6^ necrotic or live NS3 DC2.4 **(A)** 2 or **(B)** 4 weeks after the last dose. NS3-specific T cell responses were measured by IFN-γ production in an ELISpot assay. Splenocytes from vaccinated animals were re-stimulated with 3 different pools of overlapping peptides covering the complete NS3 protein (pools 1–3), or with a pool of H2^*b*^ (C57BL/6) T cell NS3 immunodominant epitopes. Plots are representative of two independent experiments (*n* = 7/group). Data represent mean SFU per 10^6^ splenocytes (±SEM). ^∗^*p* < 0.05, ^∗∗^*p* < 0.01, and ^∗∗^*p* < 0.001 (Kruskal–Wallis *H* test).

To analyze the immunogenicity of NS3 DC2.4 *in vivo*, 6–8 week old female C57BL/6 mice were immunized subcutaneously (s.c.) with 10^6^ live or necrotic NS3 DC, in a prime-boost regimen. Wild-type (WT) DC2.4 cells that do not express HCV NS3 antigen, were used as controls. Subcutaneous injection was chosen as the route of vaccination as an earlier study showed that live DC2.4 injected by this route migrated to regional LN and interacted with naïve T cells to prime Ag-specific immune response (Okada et al., 2001). This permitted a direct comparison of the immunogenicity of “standard” migrating live DC2.4 and necrotic DC2.4, expected to remain *in situ* at the site of injection. The magnitude and breadth of the NS3-specific T cell responses were analyzed by IFN-γ ELISpot at 2 (Figure 1A) and 4 (Figure 1B) weeks post-boost. ELISpots were performed by stimulating splenocytes with 3 overlapping peptide pools (approx. 30, 11–15mer peptides per pool) spanning the entire NS3 protein, and an additional pool containing only C57BL/6 immunodominant T cell peptides (which are otherwise present in Pools 1 and 3; Mikkelsen et al., 2011). Mice vaccinated with necrotic NS3 DC2.4 generated significantly higher NS3-specific IFN-γ responses than those vaccinated with live NS3 DC2.4 across all pools, at both time points (Figure 1). At 2 weeks post-vaccination the magnitude of the NS3-specific effector response induced by necrotic NS3 DC2.4 was increased by 5∼fold for Pool 1 (mean 256 SFU vs 55 SFU), 3∼fold for Pool 2 (mean 288 SFU vs 100 SFU), ∼3 fold for Pool 3 (mean 225 SFU vs 80 SFU), and almost 5 fold for the immunodominant (ID) pool (mean 332 SFU vs 72 SFU; Figure 1A). As expected, control WT DC2.4 vaccination, necrotic or live, did not elicit any NS3-specific T cell responses. At 4 weeks post-immunization, vaccination with necrotic DC2.4 induced a significantly higher NS3-specific response that was increased by approximately ∼2 fold for all peptide pools (Pool 1 mean 196 SFU vs 100 SFU; Pool 2: mean 295 SFU vs 141 SFU; Pool 3 mean 343 SFU vs 145 SFU; and ID pool 396 SFU vs 176 SFU) compared with the live DC2.4 cells (Figure 1B). Importantly as Pool 2 contains none of the immunodominant NS3 peptides and as the responses from mice vaccinated with live NS3 DC2.4 were significantly lower than the responses from mice vaccinated with necrotic NS3 DC2.4, this represents a distinct broadening of the immune response to non-dominant NS3 epitopes. Responses generated against the previously identified immunodominant T cell epitopes were enhanced and significantly higher at 2 and 4 weeks post vaccination in mice vaccinated with necrotic NS3 DC2.4 cells than those vaccinated with live NS3 DC2.4 (Mikkelsen et al., 2011). This is an important finding as strong HCV-specific T-cell responses against multiple epitopes are associated with the onset of viral clearance (Thimme et al., 2002).

### Immunization With Necrotic NS3 DC2.4 Cells Induces Multifunctional T Cell Responses and Enhances T Cell Proliferation

**FIGURE 2:**
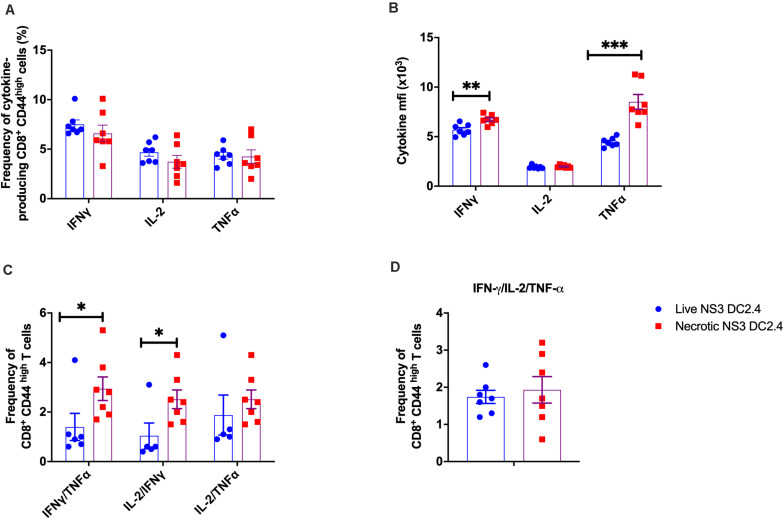
Cytokine profile of NS3-specific effector memory CD8^+^ T cells in vaccinated mice. Splenocytes from C57BL/6 mice (*n* = 7/group) vaccinated with 10^6^ live or necrotic NS3 DC2.4 cells were harvested 14 days post vaccination and restimulated with immunodominant NS3 epitopes. Intracellular cytokine staining was performed (Supplementary Figure 2) to assess the frequency of **(A)** CD3^+^ CD8^+^ T CD44 ^*high*^ effector memory T cells that are single cytokine producers of IFN-γ or IL-2 or TNF-α, **(B)** The MFI of IFN-γ, IL-2, and TNF-α producing NS3 specific CD3^+^ CD8^+^ T CD44^high^ T cells; **(C)** Frequency of CD8^+^ CD44^high^ effector memory T cells that are double cytokine producers of IFN-γ/TNF-α or IL-2/IFN-γ or IL-2-TNF-α **(D)** IFN-γ/TNF-α/IL-2 triple-positive CD8^+^ CD44 ^*high*^ effector memory T cells. Plots are representative of two independent experiments. Data show mean ± SEM. ^∗^*p* < 0.05, ^∗∗^*p* < 0.01, and ^∗∗∗^*p* < 0.001 (Mann–Whitney test).

Multifunctional T cells, simultaneously secreting IL-2, TNF-α, and IFN-γ, have been associated with the control of numerous viral infections, including HCV. Studies of HCV-infected patients proposed the importance of multifunctional CD8^+^ T cells in effective control of HCV replication and have been suggested to represent an important correlate of viral clearance in acute phase patients (Shoukry et al., 2003*;* Badr et al., 2008). Therefore to assess these responses, splenocytes from vaccinated C57BL/6 mice were harvested 2 weeks post-vaccination, stimulated *in vitro* with the immunodominant NS3 epitopes and the functional characteristics of the effector memory CD3^+^ CD44^*high*^ CD8^+^ and CD3^+^ CD44^*high*^ CD4^+^ T cells assessed by ICS (Supplementary Figure 2). CD8^+^ CD44^*high*^ effector memory T cells responded to re-stimulation by producing IL-2, TNF-α, or IFN-γ, but there was no significant difference in the frequency of single cytokine producing cells between the mice that received live or necrotic NS3 DC2.4 (Figure 2A). However, in mice vaccinated with necrotic NS3 DC2.4, cytokine secreting CD8^+^ CD44^high +^ T cells produced significantly more IFN-γ and TNF-α, but not IL-2 as shown by the mean fluorescence intensity (MFI; Figure 2B). Mice vaccinated with necrotic NS3 DC2.4 also showed a significant increase in the frequency (∼2-fold) of CD8^+^ CD44^*high*^ T cells simultaneously producing IFN-γ and TNF-α (mean percentage 2.94 vs 1.4) and IL-2 and IFN-γ (mean percentage 2.51 vs 1.04; Figure 2C). There was also a trend toward an increase in the frequency of rare, multifunctional CD8^+^ CD44^high^ T cells that produced TNF-α, IFN-γ, and IL-2 simultaneously (mean percentage 1.93.vs 1.64; Figure 2D).

**FIGURE 3:**
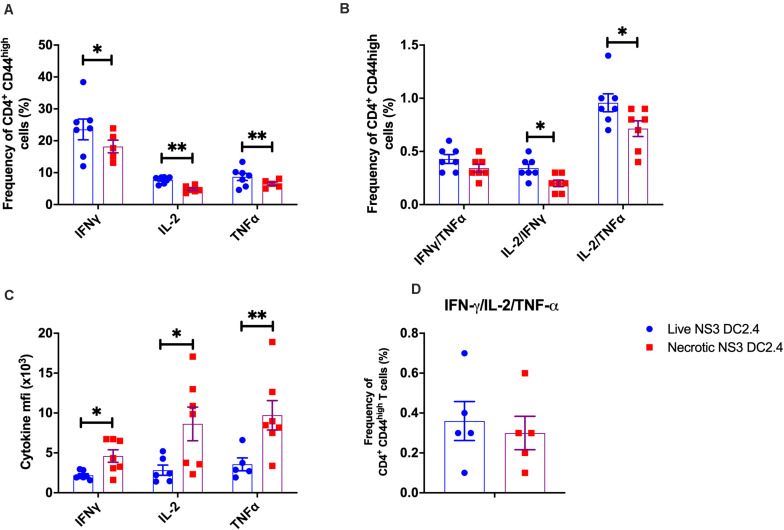
Cytokine profile of NS3-specific CD4^+^ T cells in vaccinated mice. Splenocytes from C57BL/6 mice, vaccinated with 10^6^ live or necrotic NS3 DC2.4 cells were harvested 14 days post-vaccination and restimulated with immunodominant NS3 epitopes. Intracellular cytokine staining was performed (Supplementary Figure 2) to assess the frequency of **(A)** CD3^+^ CD4^+^ T CD44^high^ effector memory T cells that are single cytokine producers of IFN-γ or IL-2 or TNF-α, **(B)** Frequency of CD4^+^ CD44^high^ effector memory T cells that are double cytokine producers of IFN-γ/TNF-α or IL-2/IFN-γ or IL-2-TNF-α. **(C)** The MFI of IFN-γ, IL-2 and TNF-α of NS3 specific CD3^+^ CD4^+^ T CD44^high^ T cells; **(D)** IFN-γ/TNF-α/IL-2 triple-positive CD4^+^ CD44^high^ effector memory T cells. Plots are representative of two independent experiments (*n* = 7/group). Data show mean ± SEM. ^∗^*p* < 0.05, ^∗∗^*p* < 0.01 (Mann–Whitney test).

Next, we examined the cytokine responses of CD4^+^ CD44^high^ effector memory T cells following vaccination with live or necrotic NS3 DC2.4 cells. Interestingly, vaccination with live rather than necrotic NS3 DC2.4 generated an increased frequency of CD4^+^ CD44^high^ T cells, secreting single cytokines IFN-γ, IL-2, or TNF-α (Figure 3A) and double cytokines IL-2/IFN-γ and IL-2/TNF-α (Figure 3B). However, there was no difference in the frequency of triple cytokine producing CD4^+^ CD44^high^ T cells between mice that received live or necrotic NS3 DC2.4 (Figure 3D). Nevertheless, although they were less frequent, cytokine producing CD4^+^ CD44^high^ T cells from mice immunized with necrotic NS3 DC2.4 cells produced significantly higher levels of IFN-γ, IL-2, and TNF-α as determined by ICS (Figure 3C). This indicates that these cells were more functionally active, as these CD4^+^ T cell-cytokines drive the maturation of protective CD8 memory responses (Shoukry et al., 2003).

**FIGURE 4:**
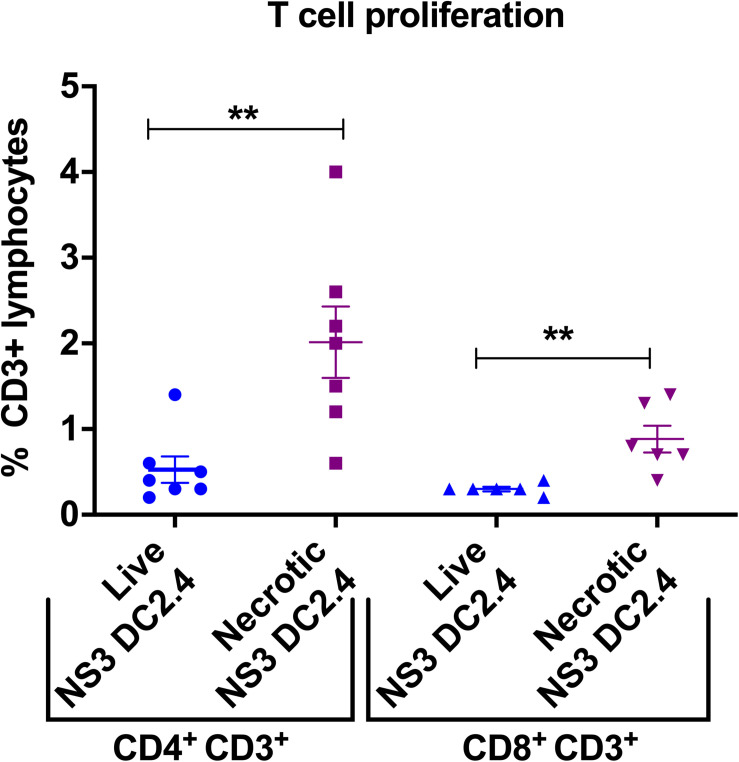
Vaccination with necrotic NS3 DC2.4 enhances *ex vivo* T-cell proliferation in vaccinated mice. Proliferation of CD4^+^ and CD8^+^ T cells harvested from splenocytes of mice vaccinated with live or necrotic NS3 DC2.4 was assessed by CFSE staining and then analyzed by flow cytometry (Supplementary Figure 3). Proliferative responses were measured by CFSE dilution. The graph demonstrates the percent proliferation of CD3^+^ CD4^+^ T cells and CD3^+^ CD8^+^T cells. Plots are representative of two independent experiments (*n* = 7/group). Data show mean ± SEM of seven samples. **p < 0.01 (Mann–Whitney test).

Robust T cell proliferation upon re-stimulation with the vaccine immunogen is the aim of any vaccine strategy. Therefore, to examine the proliferative capacity of NS3-specific T cells, induced in response to immunization with live or necrotic NS3 DC2.4 cells, splenocytes from vaccinated mice were re-stimulated with the immunodominant NS3 peptides in an *ex vivo* CFSE proliferation assay (Supplementary Figure 3). Mice vaccinated with necrotic NS3 DC2.4 cells showed a significant increase in the proliferation of NS3-specific CD8^+^ T-cells (mean percentage 0.88%) compared to mice that received live NS3 DC2.4 (mean percentage 0.3%; Figure 4), a vaccination outcome essential for effector T-cell function. Proliferation of CD4^+^ T cells from mice vaccinated with necrotic DC2.4 cells was 4-fold higher than that observed for CD4^+^ T cells from mice vaccinated with live NS3 DC2.4 (mean percentage 2% vs 0.5%; Figure 4). This is a crucial finding, as previous studies have found that a strong and sustained NS3-specific CD4^+^ T cell proliferative response is an important correlate of the clearance of acute infection and in preventing the progression to chronic HCV infection (Diepolder et al., 1995, 1997; Thimme et al., 2002; Schulze Zur Wiesch et al., 2012).

### Immunization With Necrotic NS3 DC2.4 Induces Greater Numbers and Activation of Cross-Presenting CD8a^+^ DCs in Draining Lymph Nodes

**FIGURE 5:**
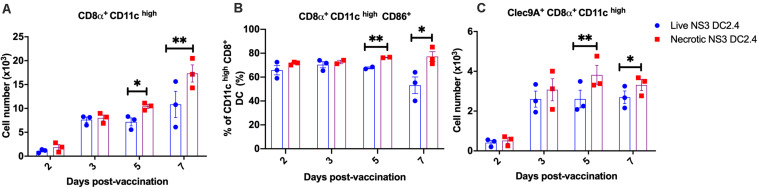
DC kinetics in DLN post vaccination. Axillary lymph nodes were harvested on days 2, 3, 5, and 7 after a single (s.c.) vaccination with 10^6^ live or necrotic NS3 DC2.4 cells CD3^–^, CD11c^*high*^, MHCII^+^ cells were identified and assessed for CD8α, CD86, and Clec9A expression. **(A)** Absolute numbers of CD11c^*high*^ MHC II^+^ CD8α^+^ DC. **(B)** Percentage of CD86 expressing CD11c^*high*^ MHC II^+^ CD8α^+^ DC. **(C)** Absolute numbers of Clec9A + CD11c^*high*^ CD8α^+^ DC. Plots are representative of two independent experiments (*n* = 5/group/time point). Data show mean ± SEM. ^∗^*p* < 0.05, ^∗∗^*p* < 0.01 (Mann–Whitney test).

Successful delivery of antigens to DC *in vivo* is crucial for effective CMI, as DC are necessary to activate naive T cells in secondary lymphoid organs (Masson et al., 2008; Pulendran and Ahmed, 2011). Therefore, to assess the mechanism by which T-cell responses were increased by vaccination with necrotic NS3 DC2.4, we investigated the kinetics of DC accumulation and activation in the LN draining the site of immunisation (DLN). We focused on CD8α^+^ DCs, due to their ability to cross-present Ag and prime CD8^+^ T cells (Shortman and Heath, 2010). Female C57BL/6 mice were vaccinated s.c. with a single dose of 10^6^ live or necrotic NS3 DC2.4 cells and axillary LNs were collected 2, 3, 5-, and 7-days post-vaccination. DC subpopulations were identified as CD3e^–^, CD11c^*high*^ MHCII^+^ and CD8a^+^ as we described previously (Supplementary Figure 4; Gargett et al., 2014; Garrod et al., 2014). The absolute numbers of cross-presenting CD8α^+^ CD11c^*high*^ DC in DLN increased over time in response to vaccination in both groups (Figure 5A). On days 5 and 7 post-vaccination, mice that received necrotic NS3 DC2.4 cells had significantly greater numbers of cross-presenting CD8α^+^ CD11c^*high*^ DC than mice that received live NS3 DC2.4 (d5: mean 10500 vs 7500 cells; d7: mean 17,300 vs 10,800 cells). The activation status of LN-resident DC, analyzed by CD86 expression, showed that mice vaccinated with necrotic NS3 DC2.4 cells displayed a significant increase in the frequency of activated CD8α^+^CD11c^*high*^ DC compared with mice vaccinated with live NS3 DC2.4 (Figure 5B).

The C-type lectin receptor, Clec9A, is expressed by CD8α^+^ DC and links the recognition of necrotic cells to the subsequent cross-presentation of dead-cell-associated antigens to T cells, thus inducing efficient CD4^+^ and CD8^+^ T cell responses *in vivo* (Sancho et al., 2008, 2009; Idoyaga et al., 2011; Zhang et al., 2012). Consequently, we assessed absolute numbers of Clec9A^+^ CD8α^+^ CD11c^*high*^ DC in DLN post vaccination. The numbers of Clec9A^+^ DC increased 2 days post vaccination in both groups of vaccinated mice (Figure 5C). However, on days 5 and 7 post-vaccination, mice that received necrotic NS3 DC2.4 cells generated significantly greater numbers of Clec9A^+^ CD11c^*high*^ CD8α^+^ DC in DLN, compared to mice vaccinated with live NS3 DC2.4 (Figure 5C). This indicates that the mechanism of enhanced immunogenicity of necrotic NS3 DC2.4 may be attributed to an increased influx of cross-presenting CD8α^+^ DC and necrosis sensing Clec9A^+^ CD8α^+^ DC, as well as an increase in activation, consistent with necrosis-enhanced cross-presentation of antigen.

### Vaccination With Necrotic NS3 DC2.4 Accelerates Clearance of NS3 Antigens From Livers of Vaccinated Mice

As liver is the main target organ for HCV infection, we examined whether immunization with necrotic NS3 DC2.4 results in enhanced clearance of HCV antigens from the liver. We previously developed a challenge mouse model (Yu et al., 2014), where DNA encoding wild type HCV NS3/4A antigens is delivered to mouse hepatocytes via hydrodynamic intravenous injection into the tail vein. As the DNA also encodes SEAP, SEAP levels in mouse serum act as a surrogate marker for intrahepatic expression of NS3/4A proteins in a minimally-invasive manner. Therefore, successful vaccination of mice should result in accelerated clearance of HCV antigen-positive hepatocytes, reflected by the accelerated clearance of SEAP from sera.

**FIGURE 6:**
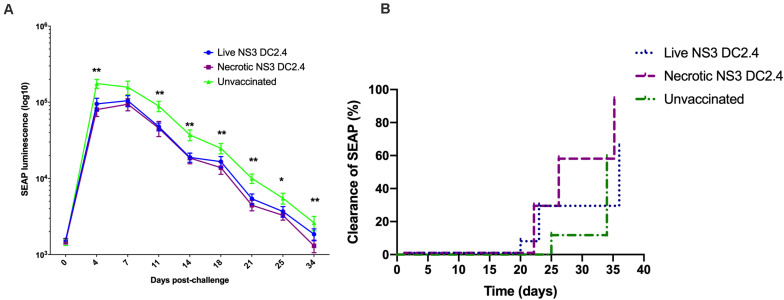
Vaccination with necrotic NS3 DC2.4 results in accelerated clearance of NS3 antigens from livers of challenged mice. Groups of C57BL6 mice were vaccinated with 10^6^ live (*n* = 15) or necrotic (*n* = 14) NS3 DC2.4 cells. Unvaccinated mice were used as controls (*n* = 17). 2 weeks post-vaccination, all groups were challenged with 20 μg NS3/4A pNFS by hydrodynamic injection. **(A)** SEAP levels were measured at 3 day intervals post-challenge in the sera of individual mice (Mann–Whitney test). **(B)** Clearance of NS3/4A antigens from the livers of challenged mice was accelerated in mice vaccinated with necrotic NS3 DC2.4 when compared to mice vaccinated with live NS3 DC2.4 and unvaccinated controls [Log-rank (Mantel-Cox) test]. Plots are representative of two independent experiments. Data show mean ± SEM. *p < 0.05, **p < 0.01.

Two groups of C57BL/6 mice were vaccinated prime-boost (s.c.) with 10^6^ live or necrotic NS3 DC2.4 cells and were challenged 10 days after the second dose with 20 μg of NS3/4A-SEAP DNA via hydrodynamic delivery. Control unvaccinated mice were also challenged. Mice vaccinated with live or necrotic NS3 DC2.4 cells had significantly lower levels of secreted SEAP, when compared to unvaccinated controls (Figure 6A). There was no significant difference in levels of SEAP between the two vaccinated groups. However, at later time points after challenge (days 18–34) mice that received necrotic NS3 DC2.4 cleared SEAP (and thus NS3 antigens from the liver) at a faster rate than mice that were vaccinated with live NS3 DC2.4 (Figure 6B). The clearance rate, determined as a SEAP level equivalent to or lower than background levels of SEAP in serum, was enhanced in mice that received necrotic NS3 DC2.4 as on day 25, 54% (8 of 14) of mice cleared SEAP to background levels, compared to only 25% (4 of 15) of mice vaccinated with live NS3 DC2.4 and 5% (3 of 17) of unvaccinated controls (Figure 6B). On day 34 post challenge, SEAP was undetected in 93% (13 of 14) of mice from the necrotic NS3 DC2.4 group, compared to 70% (10 of 15) of mice vaccinated with live NS3 DC2.4 and 67% (12 of 17) of unvaccinated controls (Figure 6B). Vaccination with necrotic NS3 DC2.4 resulted in faster clearance rate of SEAP at later time points post-challenge, indicating that vaccination with necrotic NS3 DC2.4 resulted in enhanced elimination of NS3-positive hepatocytes.

## Discussion

Despite the therapeutic cure for chronic HCV infection, a prophylactic vaccine is necessary in order to control new HCV infections and to decrease the burden of HCV-induced liver diseases, such as hepatocellular carcinoma and liver cirrhosis. An effective CMI, necessary for HCV clearance is typically of high magnitude, broad, polyfunctional, and sustained (Walker, 2010). In mice, CD8α + DCs are the main DC subset responsible for priming CMI, due to their intrinsic ability to cross-present antigens to naïve CD8^+^ T cells (Belz et al., 2004; Schnorrer et al., 2006; Dudziak et al., 2007). Therefore, CD8α^+^ DCs have been the target of many vaccine strategies, including the use of monoclonal antibodies specific for CD8α^+^ DCs to deliver Ag (Caminschi et al., 2008; Idoyaga et al., 2011; Lahoud et al., 2011).

In the past, immunization with *ex vivo* antigen loaded DC, has been used to induce immune responses against various exogenous antigens as well as tumor-associated antigens [reviewed in Palucka and Banchereau (2012)]. We took a different approach and investigated the immunogenicity of Ag positive necrotic DC as a novel vaccine strategy, since necrosis is known to promote antigen uptake, cross-presentation and activate DCs (Matzinger, 1994; Shi et al., 2003; Gamrekelashvili et al., 2015). We examined the immunogenicity of the murine DC2.4 cell line, stably transduced to express the HCV NS3 (gt1b) protein as a necrotic or live vaccine. Expression of NS3 in DC2.4 cell line had no effect on their phenotype, consistent with previous reports (Sarobe et al., 2003; Li et al., 2006; Zabaleta et al., 2008).

Following immunization with live or necrotic NS3 DC2.4 we focused our investigation primarily on the features of T cell responses that have been found to correlate with clearance of acute HCV infection in humans, including the breadth and magnitude, the proliferative capacity and multifunctionality of NS3-specific T cell responses (Diepolder et al., 1995; Missale et al., 1996). Mice vaccinated with necrotic DC2.4 were able to mount a much stronger and broader NS3-specific IFN-γ responses, directed against a greater number of different NS3 epitopes spanning the entire protein, than that observed in mice that received live NS3 DC2.4 cells, when measured 2 or 4 weeks after vaccination. In the context of a prophylactic HCV vaccine, the generation of such a strong and broad antiviral response is important as efficient production of IFN-γ has been associated with a large reduction in the viral load during acute infection in humans (Thimme et al., 2001). Additionally, analysis of the cytokine profile revealed that NS3-specific CD8^+^ and CD4^+^ effector memory T cells were able to produce IFN-γ, IL-2, and TNF-α in response to NS3 peptide stimulation. Vaccination with necrotic NS3 DC2.4 induced a greater frequency of multifunctional CD8^+^ T cells that secreted higher levels of all three cytokines, compared to vaccination with live NS3 DC2.4. Apart from their capacity for cytokine production, cytotoxic T cells are important for efficient virus control. Therefore the induction of a robust, multifunctional, CD8^+^ T cell response by necrotic NS3 DC2.4 vaccination is extremely encouraging as the appearance of such cells is associated with spontaneous recovery from acute HCV infection [reviewed by Sung et al. (2014)].

We also showed that vaccination with necrotic NS3 DC2.4 induced effector memory NS3-specific CD4^+^ T cells that secreted significantly higher levels of IFN-γ, IL-2, and TNF-α, suggesting that even though they were less frequent they were functionally more active. Especially important, as a functional CD4^+^ T cell response represents a key factor dictating the outcome of viral infection, as viral clearance is associated with rapid recall of memory T-cell responses (Shoukry et al., 2003) which depends on critical CD4 T-cell help to maintain T-cell memory (Grakoui et al., 2003) and promote maturation of memory CD8^+^ responses (Zajac et al., 1998). Vaccination with necrotic NS3 DC2.4 also resulted in enhanced (*ex vivo*) proliferation of NS3-specific CD8^+^ and CD4^+^ T cells compared to vaccination with live NS3 DC2.4. Whereas vaccination with live NS3 DC2.4 induced similar levels of proliferation for CD8^+^ and CD4^+^ T cells, vaccination with necrotic NS3 DC2.4 greatly increased the proliferation of CD4^+^ T cells. This is significant as previous studies have shown that an ongoing strong CD4^+^ proliferative response, especially that directed against NS3, was an important correlate of viral control and clearance, whereas comparable responses are absent in chronically evolving infection (Diepolder et al., 1995; Missale et al., 1996; Thimme et al., 2001).

Our study also provided information on the possible identity of DC responsible for enhanced T cell responses *in vivo*, as there are no direct means by which genotype 1b NS3 antigen can be detected in cross-presenting DC. Vaccination with necrotic NS3 DC2.4 resulted in increased numbers of activated CD8α^+^ CD11c^*high*^ DCs in the DLN, a cell population that is essential for cross-presentation of antigens derived from dying cells to naive CD8^+^ T cells. It is likely that this effect is mediated through Clec9A signaling via F-actin (Zhang et al., 2012) as necrotic NS3 DC2.4 vaccination increased the absolute numbers of Clec9A^+^ CD8α^+^ CD11c^*high*^ DCs in DLN. Targeting antigens to Clec9A–expressing DC is a potential mechanism to enhance vaccine efficiency, as targeting Clec9A DC with a monoclonal antibody induced strong HIV gag-specific Th1 and CD8^+^ T-cell responses in mice (Idoyaga et al., 2011).

To examine the ability of vaccine-induced immune cells to migrate to the liver, we performed experiments to address the *in vivo* function of the immune response. We used a hydrodynamic challenge model, developed in our laboratory that results in NS3/4A expression in hepatocytes and secretion of SEAP in serum (Yu et al., 2014). As expected, vaccination with live and necrotic NS3 DC2.4 resulted in lower SEAP levels in serum, compared to that of unvaccinated controls. At later time points necrotic NS3 DC2.4 vaccination resulted in accelerated NS3 clearance from the liver. It is plausible that CTLs primed by necrotic cell vaccination migrated to the liver and cleared NS3-expressing hepatocytes more efficiently. A major limitation of this model is that it is necessary to target 5–10% of hepatocytes to detect SEAP in serum. Thus, no HCV vaccine candidate to date, in our laboratory, has been able to completely prevent NS3/4A antigen expression in hepatocytes. In contrast, during natural HCV infection, it is likely that only a few cells get infected initially. Nevertheless, due to the lack of available HCV challenge options in mice, it is still a viable model to assess the rate of clearance of viral antigens, particularly as the migration of immune cells to the liver of an infected individual is likely to be vital in the outcome of infection with HCV, in order to tip the balance in favor of the host.

Many factors influence the cross-presenting efficiency of DC, which is not only dependent on the ability to capture Ag, but is also affected by critical factors such as the form and quantity of Ag available, the route of Ag uptake, Ag degradation in endosomal-lysosomal compartments and Ag entry into the major histocompatibility complex class I (MHC-I) pathway (Burgdorf et al., 2007; Lin et al., 2008; Amigorena and Savina, 2010). The immunogenicity of freeze-thawed necrotic cells was assessed previously and several studies failed to show that necrotic cells generated Ag-specific CD8^+^ T cell mediated immune responses (Scheffer et al., 2003; Bartholomae et al., 2004; Yoon et al., 2008; Gamrekelashvili et al., 2012). To the best of our knowledge, our study represents the first report that clearly demonstrates enhanced immunogenicity of necrotic cells loaded with viral antigen and used as a prophylactic vaccine. Our findings are supported by those of a previous study, which found that the induction of necrosis by heating the cells induced Ag-specific T cell activation both *in vitro* and *in vivo*, using ovalbumin as a model antigen (Gamrekelashvili et al., 2013). That study found that inactivation of DPP-3 and TOP-1 peptidases by heating resulted in protection of their oligopeptide substrates from degradation and accounted for the mechanism by which heat-induced necrotic cells activated Ag-specific T cell responses. Furthermore, in contrast to freeze-thawing, heat-induced necrosis generates extensive antigen denaturation and aggregation, which should result in more efficient processing and presentation of antigens by DC.

In a natural HCV infection, since HCV is tissue tropic and non-cytopathic, cross-presentation of viral antigens is likely to be limited. Vaccination with necrotic cells overcomes these limitations as it can direct the NS3 antigen into the MHC-I/cross-presenting pathway and thus may constitute an exciting new prophylactic vaccine.

## Data Availability Statement

The original contributions presented in the study are included in the article/Supplementary Material, further inquiries can be directed to the corresponding author.

## Ethics Statement

The animal study was reviewed and approved by Women’s and Children’s Health Network and the University of Adelaide Animal Ethics Committee.

## Author Contributions

ZM and MM performed experiments and contributed to data analysis, data evaluation, and manuscript completion. WY performed the hydrodynamic challenge and assisted in experiments. BG-B designed and performed experiments and wrote the manuscript. JG, DW, and ZA-D assisted in experiments. JT contributed to data evaluation and manuscript completion. EG contributed to experimental planning, data evaluation, and completion of the manuscript. All authors contributed to the article and approved the submitted version.

## Conflict of Interest

The authors declare that the research was conducted in the absence of any commercial or financial relationships that could be construed as a potential conflict of interest.

## Footnotes

1 http://www.beiresources.org/Catalog/BEIPeptideArrays/NR-3742.aspx
