# Supplementary material for: Enhanced T Cell Responses Induced by a Necrotic Dendritic Cell Vaccine, Expressing HCV NS3
Source: Front Microbiol. 2020 Nov 24;11:559105. doi: 10.3389/fmicb.2020.559105 (PMC7739890; doi:10.3389/fmicb.2020.559105)
Supplement: Supplementary file 1 [file Image_1.pdf]

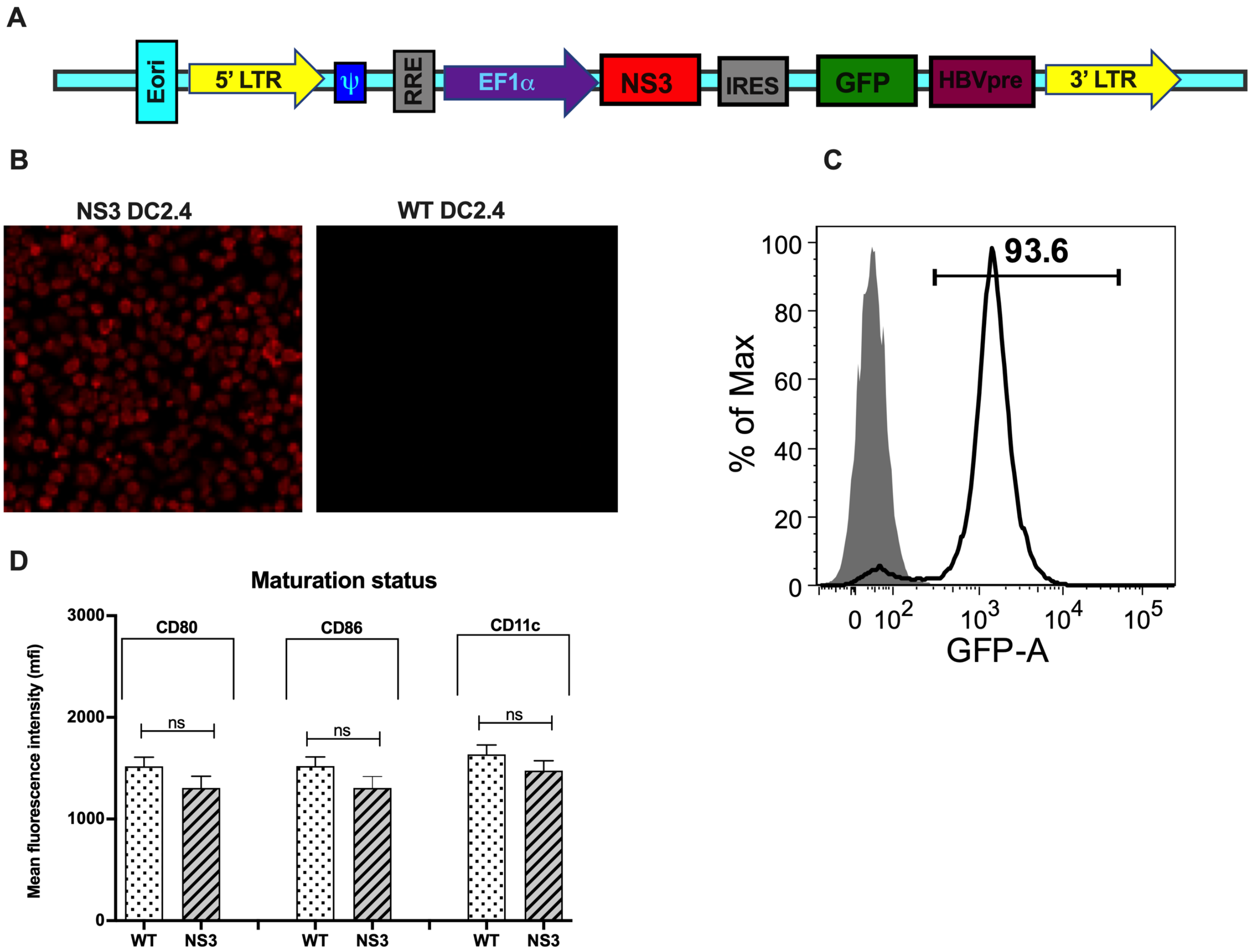

Supplementary Figure 1. Generation of a stable DC2.4 cell line expressing HCV gt1b NS3 protein. (A) Third-generation lentiviral vectors overexpressing codon optimized HCV NS3 were created with Gateway technology, where the codon optimised NS3 (gt1b) was inserted under the control of the EF-1α promoter and the marker gene (GFP) was expressed from IRES. (**B**) NS3 expression in NS3 DC2.4 stable cells was confirmed by immunofluorescence **(C)** GFP expression was used to confirm the purity of stable NS3 DC2.4 cell line by flow cytometry (black line), against wild type DC2.4 used as controls (grey). **(D)** Flow cytometric analysis of WT DC2.4 and NS3 DC2.4 cells stained for CD11c, CD80 and CD86.
