# Supplementary material for: Enhanced T Cell Responses Induced by a Necrotic Dendritic Cell Vaccine, Expressing HCV NS3
Source: Front Microbiol. 2020 Nov 24;11:559105. doi: 10.3389/fmicb.2020.559105 (PMC7739890; doi:10.3389/fmicb.2020.559105)
Supplement: Supplementary file 2 [file Image_2.pdf]

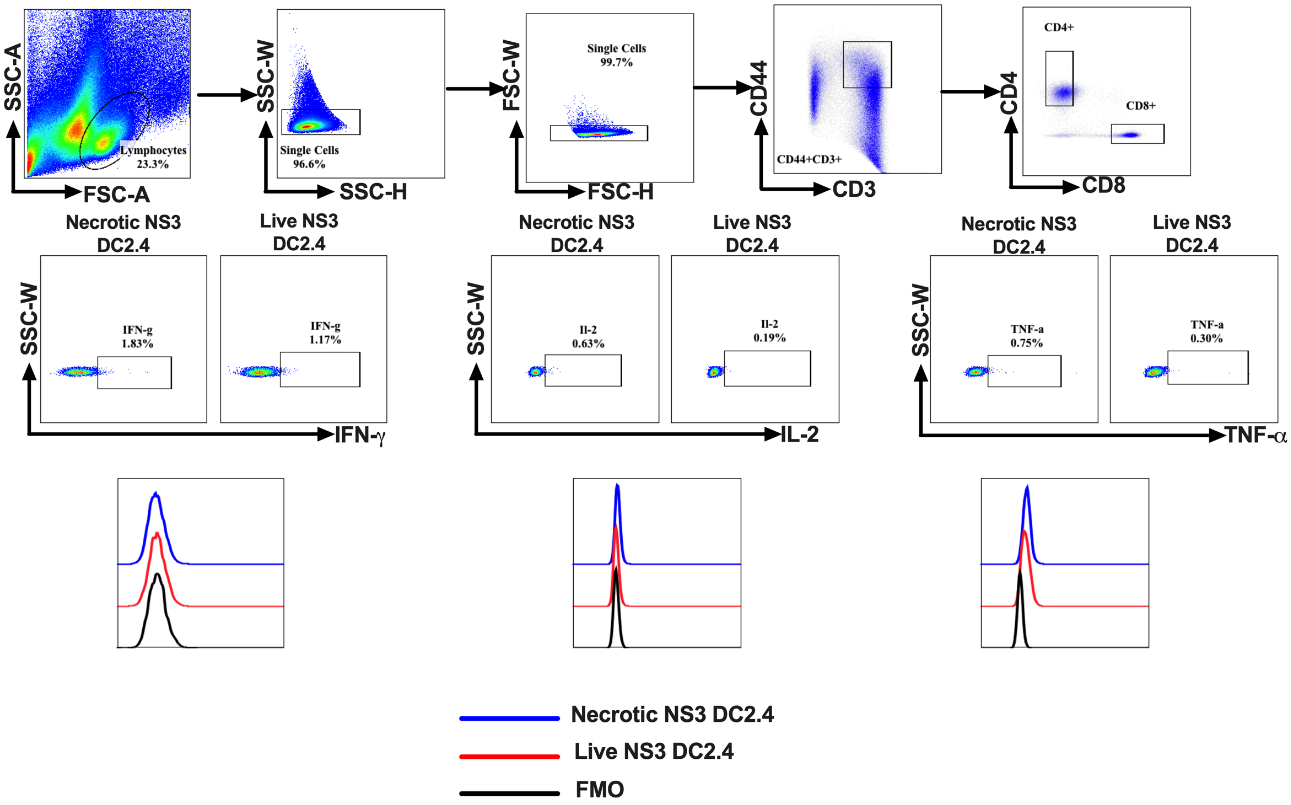

Supplementary Figure 2: Gating strategy used to detect cytokine producing CD8^{+} and CD4^{+} T cells in vaccinated mice. Splenocytes from C57BL/6 mice (n=7/group) vaccinated with 10^6 live or necrotic NS3 DC2.4 cells were harvested 2 weeks post-vaccination, stimulated with the immunodominant NS3 epitopes and the functional characteristics of the effector memory CD3^{+} CD44^{high} CD8^{+} and CD3^{+} CD44^{high} CD4^{+} T cells assessed by ICS. Splenocytes were gated on the lymphocyte population, followed by doublet discrimination, and then gated on CD3^{+} CD44^{+} cells and finally CD4^{+} or CD8^{+} cells. Representative plots for IL-2, TNF-α and IFN-γ -positive cells are shown.
