# Supplementary material for: Enhanced T Cell Responses Induced by a Necrotic Dendritic Cell Vaccine, Expressing HCV NS3
Source: Front Microbiol. 2020 Nov 24;11:559105. doi: 10.3389/fmicb.2020.559105 (PMC7739890; doi:10.3389/fmicb.2020.559105)
Supplement: Supplementary file 3 [file Image_3.pdf]

frontiers

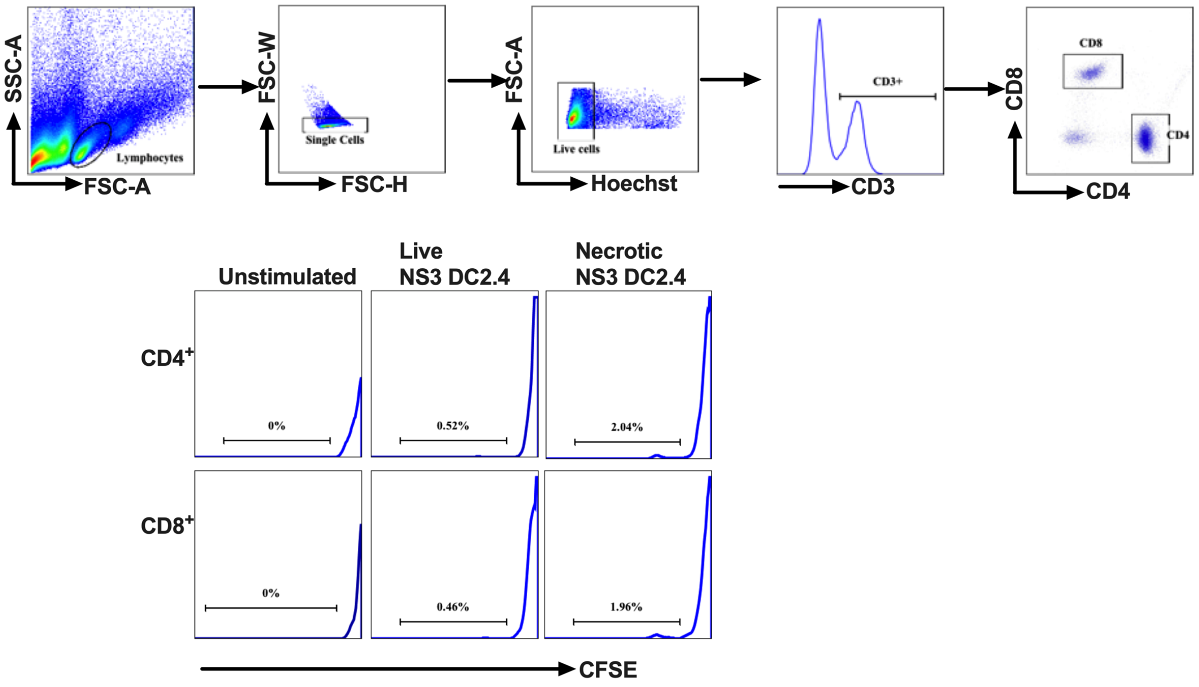

Supplementary Figure 3: Proliferation of $CD8^+$ and $CD4^+$ T cells was assessed by flow cytometry after CFSE staining. Splenocytes from vaccinated mice were labelled with 10 µM CFSE (CellTrace CFSE Cell Proliferation Kit protocol; Life Technologies) as per the manufacturer's instructions and stimulated *in vitro* with 4µg/mL of immunodominant NS3 peptides or left unstimulated. After 5 days, CFSE-labelled splenocytes were surface stained with CD3-PerCP-Cy5.5, CD4-eFluor450 and CD8-APC-Cy7 followed immediately by Hoechst staining prior to analysis by flow cytometry. Proliferative responses were measured by CFSE dilution assay of cell proliferation. Representative plots of gating strategy and CFSE dilution are shown.
