# Supplementary material for: Enhanced T Cell Responses Induced by a Necrotic Dendritic Cell Vaccine, Expressing HCV NS3
Source: Front Microbiol. 2020 Nov 24;11:559105. doi: 10.3389/fmicb.2020.559105 (PMC7739890; doi:10.3389/fmicb.2020.559105)
Supplement: Supplementary file 4 [file Image_4.pdf]

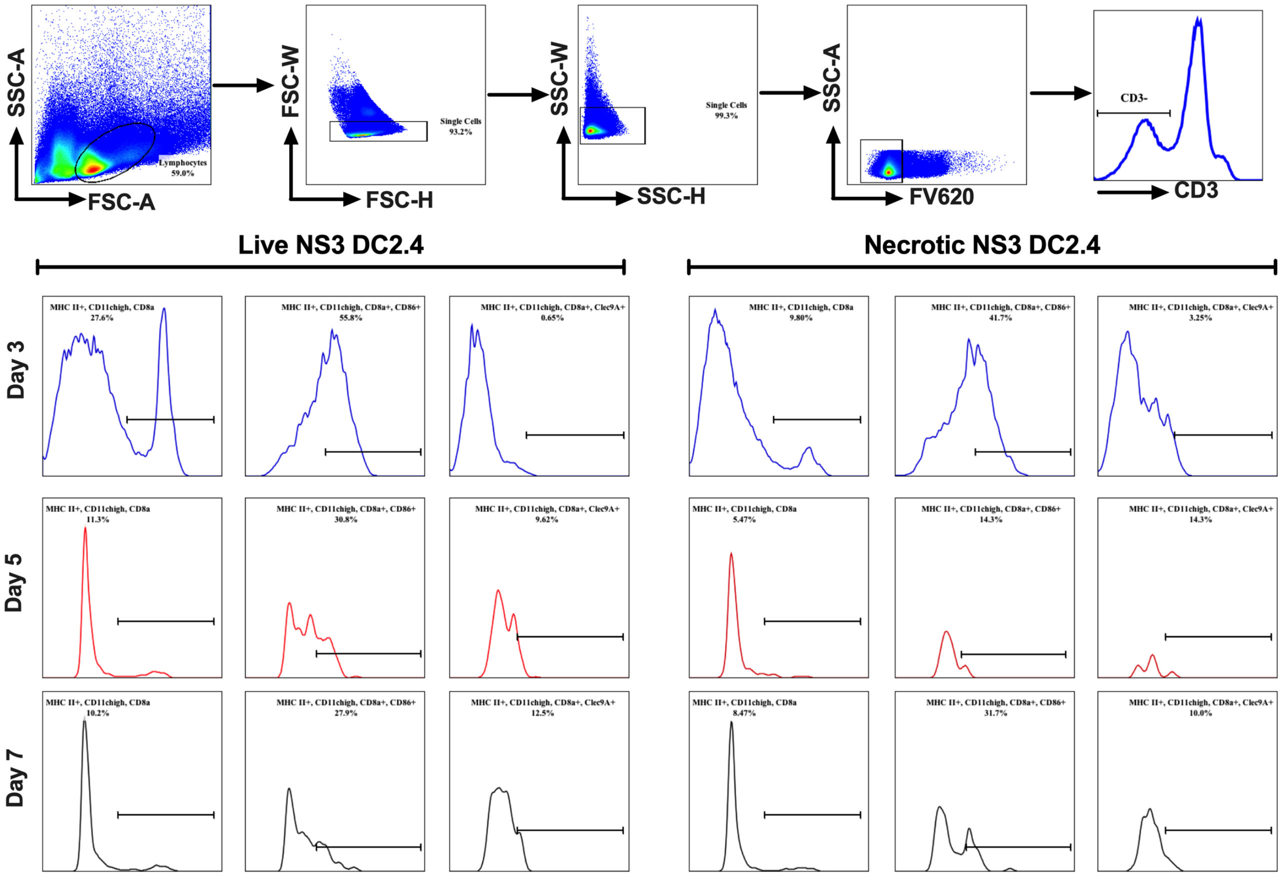

Supplementary Figure 4: Gating strategy of DC isolated from DLN post vaccination. Axillary lymph nodes were harvested on days 2, 3, 5 and 7 after a single (s.c.) vaccination with 10^6 live or necrotic NS3 DC2.4 cells. DC staining was performed on cell suspensions using CD8a-APC-Cy7, CD11c-PeCy7, CD86-PE, MHCII-FITC and Clec9A-PE antibodies. Purified lymphocytes cells were double discriminated using FSC and SSC followed by discrimination of live cells and then gated on $CD3^-$ (non-T) cells followed by $CD11c^{high}$ MHC-II^+ cells (DC), $CD11^{chigh}$ MHC-II^+ $CD8a^+$ cells (cross-presenting $CD8a^+$ DC) and $CD11c^{high}$ $MHCII^+$ $CD8a^+$ $Clec9A^+$ (necrosis-sensing $Clec9A^+$ DC) or $CD11c^{high}$ $MHCII^+$ $CD8a^+$ $CD86^+$ (activated cross presenting $CD8a^+$ DC). Gating strategy and representative plots from day 3, 5 and 7 are shown.
